# Innovative approaches to establish and characterize primary cultures: an *ex vivo* 3D system and the zebrafish model

**DOI:** 10.1242/bio.022483

**Published:** 2016-11-28

**Authors:** Chiara Liverani, Federico La Manna, Arwin Groenewoud, Laura Mercatali, Gabri Van Der Pluijm, Federica Pieri, Davide Cavaliere, Alessandro De Vita, Chiara Spadazzi, Giacomo Miserocchi, Alberto Bongiovanni, Federica Recine, Nada Riva, Dino Amadori, Ennio Tasciotti, Ewa Snaar-Jagalska, Toni Ibrahim

**Affiliations:** 1 Osteoncology and Rare Tumors Center, Istituto Scientifico Romagnolo per lo Studio e la Cura dei Tumori (IRST) IRCCS, via P. Maroncelli 40, Meldola 47014, Italy; 2 Leiden University Medical Center, Department of Urology, J-3-100, Albinusdreef 2, Leiden 2333ZA, The Netherlands; 3 Department of Molecular Cell Biology, Institute of Biology, Leiden University, Sylviusweg 72, Leiden 2333BE, The Netherlands; 4 Pathology Unit, Morgagni-Pierantoni Hospital, Forlì 47121, Italy; 5 Unit of Surgery and Advanced Oncologic Therapies, Morgagni-Pierantoni Hospital, Forlì 47121, Italy; 6 Department of Regenerative Medicine, Houston Methodist Research Institute, 6670 Bertner Avenue, Houston, TX 77030, USA

**Keywords:** Primary cultures, Collagen scaffolds, Zebrafish, Near-patient models

## Abstract

Patient-derived specimens are an invaluable resource to investigate tumor biology. However, *in vivo* studies on primary cultures are often limited by the small amount of material available, while conventional *in vitro* systems might alter the features and behavior that characterize cancer cells. We present our data obtained on primary dedifferentiated liposarcoma cells cultured in a 3D scaffold-based system and injected into a zebrafish model. Primary cells were characterized *in vitro* for their morphological features, sensitivity to drugs and biomarker expression, and *in vivo* for their engraftment and invasiveness abilities. The 3D culture showed a higher enrichment in cancer cells than the standard monolayer culture and a better preservation of liposarcoma-associated markers. We also successfully grafted primary cells into zebrafish, showing their local migratory and invasive abilities. Our work provides proof of concept of the ability of 3D cultures to maintain the original phenotype of *ex vivo* cells, and highlights the potential of the zebrafish model to provide a versatile *in vivo* system for studies with limited biological material. Such models could be used in translational research studies for biomolecular analyses, drug screenings and tumor aggressiveness assays.

## Abstract (precis)

**Summary:** Patient-derived primary cells maintain their original phenotype when cultured on 3D scaffolds, and their *in vivo* behavior can be effectively studied with innovative zebrafish models.

## INTRODUCTION

Tissue specimens from patients provide the ideal experimental material to study the heterogeneous biology and behavior of cancer cells. Conversely, immortalized cell lines undergo a phenotypic, epigenetic and sometimes genetic drift from the early matching tumor, maintaining only part of their pathologically relevant properties (Williams et al., 2013). However, the number of research experiments that can be carried out on primary tissue specimens is often extremely limited due to the often small amount of material available from these samples. The establishment of *in vivo* animal models required to evaluate and validate a number of cancer cell phenotypes is thus not always feasible. In addition, many of the features that characterize cancer cells may be lost in the conventional systems used in *in vitro* studies up to now, limiting our understanding of disease progression and our potential to screen for effective drugs (Allen and Jones, 2011; Dvorak et al., 2011; Hanahan and Weinberg, 2011). Biomimetic, tridimensional (3D) models represent an optimal tool to provide a tissue-like context for cell cultures. These systems mimic the composition and signaling cues of the tumor extracellular matrix, which may influence the genotype, phenotype and behavior of cancer cells (Lamhamedi-Cherradi et al., 2014; Yamada et al., 2007). For this reason the use of 3D culture has been extensively explored for the study of cancer cell lines (Florczyk et al., 2013; Hirt et al., 2015; Fitzgerald et al., 2015) and has been reported also for patient-derived primary material (Reagan et al., 2014; Lv et al., 2016). These culture systems, demonstrating greater fidelity to the *in vivo* scenario, emerged as valuable approaches to investigate the tumor cell biology and to screen new drugs. Despite their advantages, i.e. the possibility of finely controlling culture conditions and of optimizing scaffold structure and composition, these approaches cannot reproduce the complex environment of an *in vivo* model. Conversely, the zebrafish could provide a rapid and effective *in vivo* means of screening for functional cancer-related phenotypes, and cell migratory and invasive abilities starting from limited primary material. The zebrafish system is currently being evaluated in relation to its potential implications for personalized therapy, and studies on human cancer xenografts in this model are steadily increasing (Barriuso et al., 2015). However, there is still relatively little information in the literature on the engraftment of near-patient material (or patient-derived xenografts, PDX). Some studies have reported the successful engraftment of near-patient specimens, i.e. cell suspensions or tissue fragments, in zebrafish, especially in its embryonic stage. This includes specimens of healthy and fibrotic tissue (Benyumov et al., 2012; Orlova et al., 2014) as well as malignant tissue from various sources such as gastrointestinal tumors (Marques et al., 2009), prostate cancer (Bansal et al., 2014), glioblastoma multiforme (Rampazzo et al., 2013) and leukemia (Bentley et al., 2015; Pruvot et al., 2011). In the field of cancer research, rare tumors represent a heterogeneous subset of malignancies whose natural biology, treatment and potential clinical outcome may differ significantly (Raghavan et al., 2012). Their heterogeneity and low incidence also hamper near-patient research studies. Liposarcoma is the most common soft-tissue sarcoma, accounting for up to 15% of all cases of adult sarcoma (Crago and Singer, 2011). It is divided into three different subtypes on the basis of histological characteristics: well differentiated/dedifferentiated (WDLPS/DDLPS), myxoid (MLPS) and pleomorphic (PLS) (Clark et al., 2005; Conyers et al., 2011; Dei Tos, 2000; Dodd, 2012; Fletcher, 2014). Complete surgical resection represents the standard medical care for patients with localized disease, while chemotherapy and radiotherapy for metastatic or unresectable tumors is preferred but still much debated. In recent years, significant advances have been made in our understanding of the molecular and cellular biology of WDLP and DDLP, opening up new avenues of research for the diagnosis and treatment of these patients. Chromosomal amplification of the 12q13–15 region, including the *MDM2* and *CDK4* genes, is the hallmark genetic change in these diseases. Amplification of *MDM2* occurs in almost all DDLPS cases and detection by fluorescence *in situ* hybridization is widely used as a diagnostic tool (Hoffman et al., 2011; Hostein et al., 2004; Rieker et al., 2010). However, the extremely variable biology of these tumors has limited the identification of predictive and prognostic biomarkers, and also hindered the collection of accurate data on DDLPS response to chemotherapy (Italiano et al., 2012; Tseng et al., 2013). Doxorubicin and ifosfamide represent the standard chemotherapy agents for the treatment of advanced soft tissue sarcoma, objective response rates (ORRs) ranging from 15-30% when the drugs are used singly and from 20-40% when they are administered in combination, with no difference in overall survival (Edmonson et al., 1993). Recently, trabectedin was approved for the treatment of advanced soft tissue sarcoma after it was shown to reduce the risk of disease progression or death by 45% compared to dacarbazine, with an ORR of 9.9% (Demetri et al., 2016).

We used 3D collagen-based scaffolds and a zebrafish model to study the invasive ability, chemotherapy sensitivity and biomarker expression of near-patient cells derived from a dedifferentiated liposarcoma lesion, and to evaluate the translational potential of these experimental models.

## RESULTS

### Establishment of the *ex vivo* 3D tumor model

**Fig. 1.**
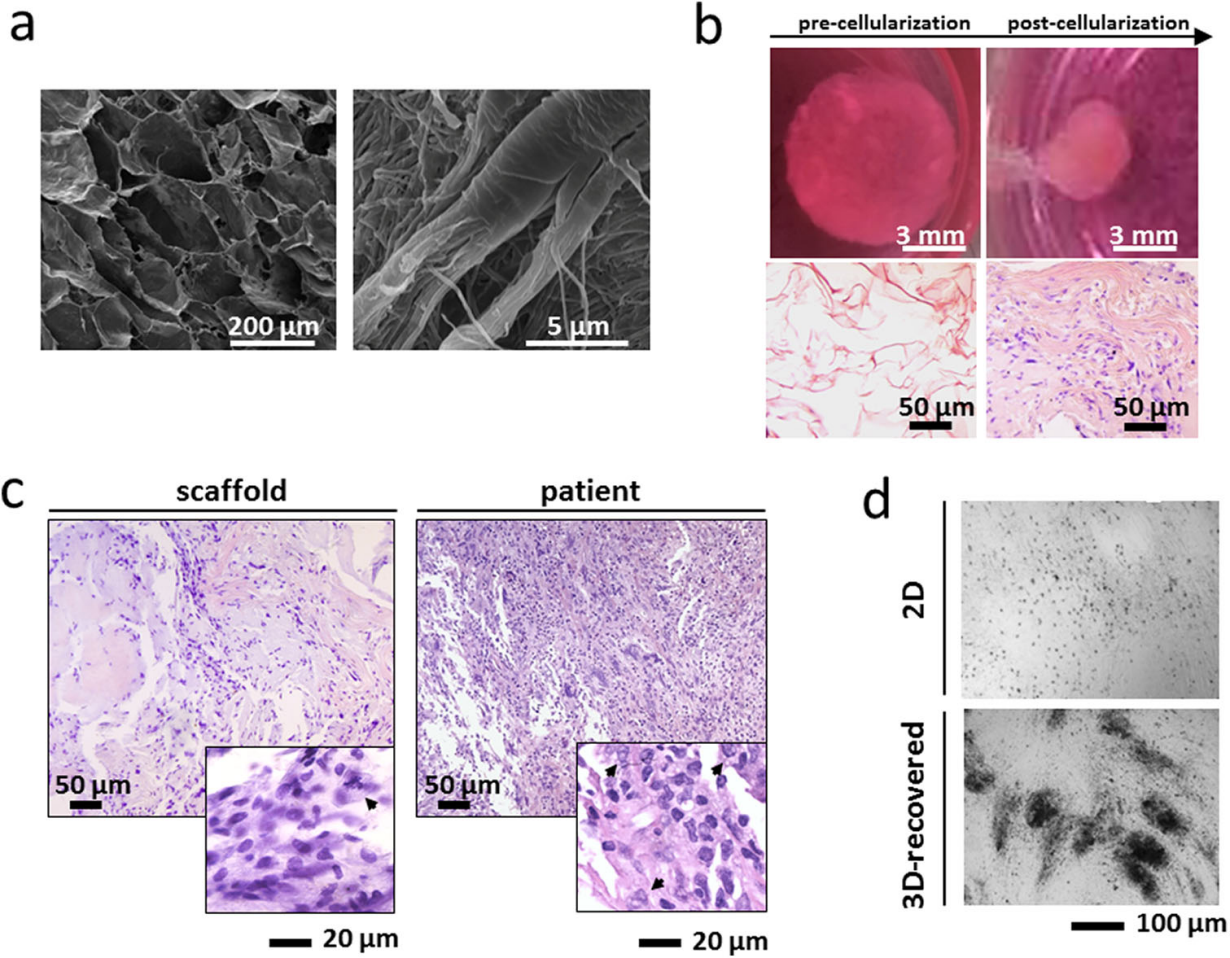
**Characterization of the *ex vivo* 3D tumor model.** (A) Scanning electron microscopy (SEM) analysis of collagen-based scaffolds at different magnifications. Images were taken with a FEI Nova NanoSEM microscope. (B) Pictures of collagen scaffolds pre- and post-cellularization with primary liposarcoma cells and hematoxylin & eosin staining of paraffin-embedded sections of the scaffold pre- and post-cellularization (C) Hematoxylin & eosin staining of paraffin-embedded sections of 3D scaffolds cultured with primary liposarcoma cells and of the histological specimen. Arrowheads indicate tumor cells. (D) Inverted microscopy pictures of 2D-cultured and 3D-recovered liposarcoma cells.

The scaffolds displayed an average porosity of 85%, with a mean pore size of 24.674×10^3^ (±1.332) µm^2^ and a total void space of 48.901×10^3^ (±0.218) μm^3^ (Fig. 1A). Hematoxylin and eosin (HE) staining of the surgical specimen showed a tumor with high cellularity and mostly spindle-shaped cells arranged in storiform patterns. Focal transition into well differentiated atypical adipose tissue was present. The primary culture obtained from the surgical material was stable for 4-5 subculture passages in both the collagen-based scaffolds and monolayer cultures. The biomimetic properties of the collagen scaffold induced primary cells to strongly interact with the surrounding matrix. After 3 weeks of culture the scaffolds appeared completely remodeled, showing increased density and altered geometry: the macroscopic dimensions changed from 1×9 mm to about 3×3.5 mm and the matrix appeared markedly denser with a reorganization of the collagen fibers (Fig. 1B). Although the lower cellularity, the scaffold sections showed a tissue-like organization with features similar to that of the patient's tumor. In particular the matrix structure and the morphology of cancer cells with large and polylobate nuclei (arrowhead in Fig. 1C) were maintained, as reviewed by an experienced pathologist in sarcoma (Fig. 1C). This architecture was completely lost in monolayer systems. Finally, 3D scaffold culturing promoted cell-to-cell adhesion, resulting in enhanced cell aggregation after recovery of primary cells from the constructs (Fig. 1D).

### Cancer cell enrichment and preservation of liposarcoma-associated markers in the 3D tumor model

**Fig. 2.**
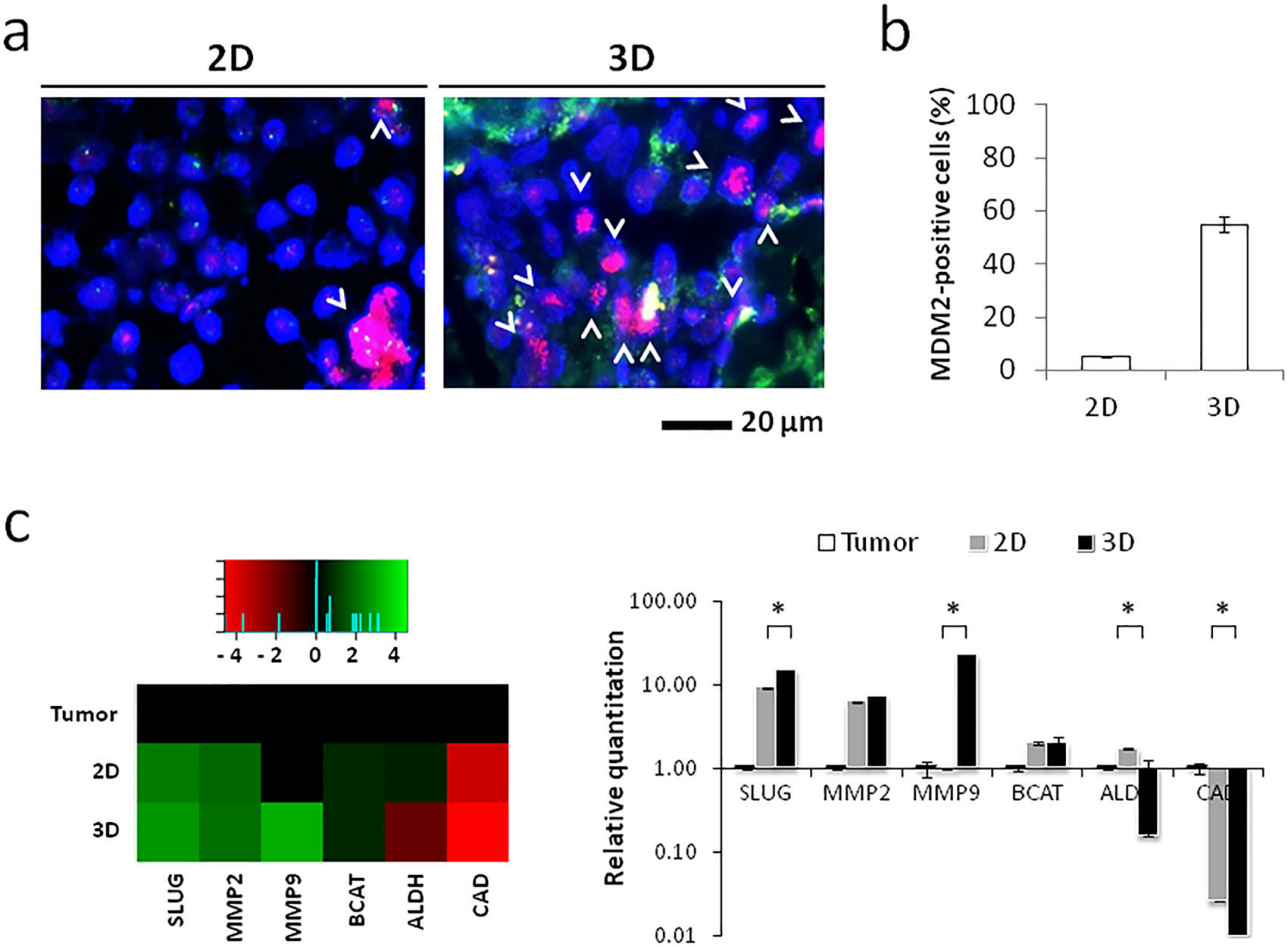
**Liposarcoma cells enrich in the 3D tumor model.** (A) Fluorescence *in situ* hybridization for *MDM2* amplification in 2D and 3D cultured liposarcoma cells. Arrowheads represent the cells with *MDM2* amplification. (B) Percentages of cells positive for *MDM2* amplification in 2D and 3D cultures. Data are mean±s.d. (*n*=3). (C) Heatmap representation and relative quantitation values of Slug (SLUG), beta-catenin (BCAT), matrix metalloproteinase 2 (MMP2), matrix metalloproteinase 9 (MMP9) and aldehyde dehydrogenase 1 (ALDH) in 2D and 3D cultured primary liposarcoma cells and in the patient's original tumor specimen. Data are mean±s.d. (*n*=3). Unpaired *t*-test between 2D and 3D relative quantitation values, *P=0.0063 for SLUG, *P=0.092 for CAD, *P=0.0114 for MMP9, *P=0.0026 for ALDH.

The presence of tumor cells in primary cultures was confirmed by *MDM2* *in situ* hybridization of paraffin-embedded scaffold sections and of cytospin slides of monolayer cells. Culturing within our 3D system conferred a selective advantage to cancer cells with respect to their stromal counterpart which resulted in a strong enrichment of dedifferentiated liposarcoma cells from fibroblasts (Fig. 2A). The percentage of cells harboring *MDM2* amplification in monolayer cultures was significantly lower than that of cells cultured on collagen-based scaffolds, i.e. *<*5% and >50%, respectively (Fig. 2B). Furthermore, cells in our 3D model showed an induction of specific biomarkers associated with liposarcoma pathogenesis, e.g. β-catenin and E-cadherin, and aggressiveness, e.g. ALDH1, MMP2, MMP9 and Slug. The expression of β-catenin, MMP2, MMP9 and Slug was significantly higher in 3D-cultured cells compared to cells in monolayer cultures, while E-cadherin levels were significantly reduced. Conversely, ALDH1 expression was higher in monolayer cultures than in the 3D model (Fig. 2C).

### Efficacy of chemotherapy in 3D-enriched liposarcoma cells

**Fig. 3.**
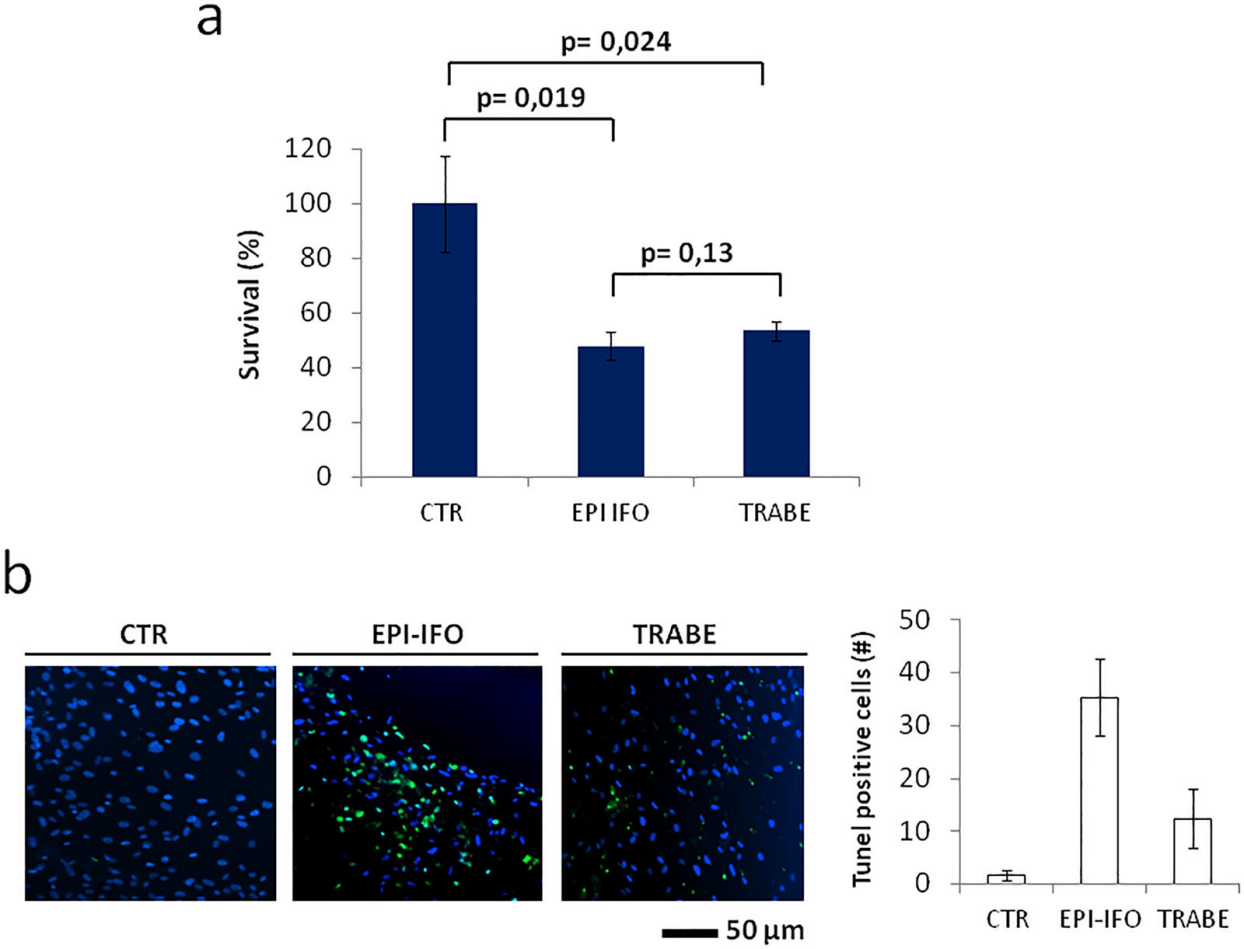
**Sensitivity of primary liposarcoma cells to chemotherapy drugs.** (A) Survival percentages of primary liposarcoma cells not treated (CTR), treated with epirubicin plus ifosfamide (EPI IFO) or treated with trabectedin (TRABE). Data are mean±s.d. (*n*=5). Unpaired *t*-test. (B) TUNEL staining of primary liposarcoma cells CTR, EPI IFO, TRABE (green, TUNEL positive cells; blue, nuclei stained with DAPI). Images were analyzed with Image J software (NIH Image, Bethesda, MD). Percentages of TUNEL-positive primary liposarcoma cells not treated, treated with epirubicin plus ifosfamide or treated with trabectedin. Data are mean±s.d. (*n*=5).

We assessed the sensitivity of our primary liposarcoma culture to the combination of epirubicin plus ifosfamide (standard chemotherapy for DDLPS), and also to trabectedin. Drugs were tested on 3D-enriched cancer cells to assess sensitivity in a tumor-representative population. Both tested schedules showed a strong antiproliferative effect on DDLPS cells, with survival percentages significantly lower than in untreated controls for both the combination of epirubicin plus ifosfamide and trabectedin alone (48% and 53%, respectively) (Fig. 3A). Drug efficacy was confirmed by TUNEL assessment of apoptosis. The number of apoptotic cells counted in the presence of either treatment was significantly higher than that of untreated controls (Fig. 3B). TUNEL positivity resulted higher in cells treated with epirubicin plus ifosfamide compared to trabectedin alone (Fig. 3B).

### Engraftment and invasive ability of primary cells in the zebrafish model

**Fig. 4.**
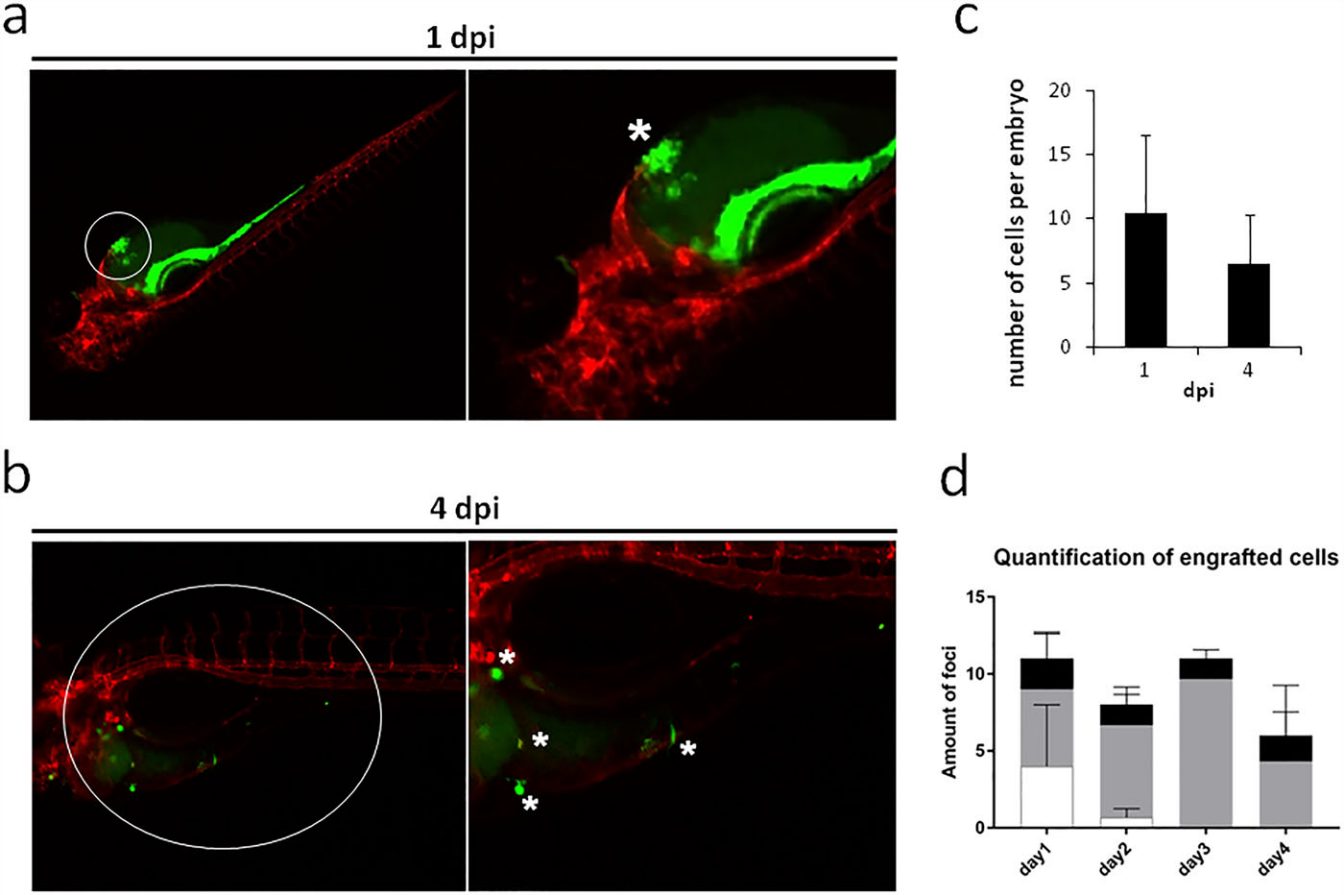
**Representative stereo micrograph images of primary liposarcoma cells (green, CFSE) injected into 2 dpf *Tg(Kdrl:mCherry)* zebrafish embryos.** Images taken at (A) 1 dpi and (B) 4 dpi. White circles indicate the area zoomed in the close-ups; white asterisks indicate the injected cells (A) and the invading cells (B). The number of engrafted foci per embryo is reported. Quantification of total number of engrafted foci at 1 and 4 dpi (C) (mean±s.d., *n*=4) and quantification of engrafted foci in the three different anatomical regions of the zebrafish embryos (D) (mean±s.d., *n*=3). White, tail region; gray, body region; black, head region. At 4 dpi, we detected that liposarcoma-derived cells survived *in vivo* and spread from the injection site. Of note, injected embryos showed an aspecific CFSE signal in the gastrointestinal trait of the embryos, at the initial timepoint of the experiment (1 dpi), due to dye leakage. This aspecific signal faded out at the later timepoint, as visible in the pictures of 4 dpi embryos.

Given the low amount of cells available, we injected cells resuspended in Matrigel, in order to favor the engraftment whilst maintaining cell-to-cell contact. We successfully engrafted cells into Tg(*kdrl*:mCherry) zebrafish using Matrigel as a vehicle and implanting the cells into the embryonic heart cavity at 2 day post-fertilization (dpf). The implantation allowed cells (asterisk in Fig. 4A) to be contained within the embryonic heart cavity whilst retaining close proximity to anatomical vasculature and the epithelium surrounding the yolk sac, permitting adhesion and potentially active migration of cancer cells (Fig. 4A). The zebrafish model, used primarily to stabilize the near-patient material *in vivo* and to assess the invasive capacity of cancer cells, permitted the tracking of single-cell movement across tissues of the developing zebrafish embryos. Cells were shown to be scattered throughout the implantation area (asterisks in Fig. 4B) at 4 day post-implantation (dpi), confirming local invasiveness ability (Fig. 4B). The cells recapitulated their polarized phenotype *in vivo* and remained visible with CFSE labeling for the entire duration of the *in vivo* experiments. The implanted cells survived in the zebrafish embryos over time: the average number of foci of engrafted cells per embryo was about 10.5 at 1 dpi and about 6.5 at 4 dpi (Fig. 4C). In order to detect the preferential site of cell engraftment, the imaged embryos were divided into three anatomical regions (head, body and tail, Fig. S1) and the number of foci of engrafted cells in each region at every time point were counted. As shown by the graph in Fig. 4D, the cells engrafted preferentially in the body region of the embryos, and some foci engrafted in the head region, while cells in the tail region tended not to survive at later time points.

## DISCUSSION

In the present study we developed a highly translational approach that took advantage of both *in vitro* 3D cultures and the *in vivo* zebrafish model to study a patient-derived surgical specimen. This strategy allowed for an effective molecular and functional characterization of the near-patient primary culture. In recent years, increasing attention has been paid to 3D culture systems which enable cells to retain more *in vivo*-like biological behavior than cultures grown in bidimensional substrates (Fischbach et al., 2007). Whilst this feature is extremely useful for the culture of established cell lines, it becomes fundamental when dealing with patient-derived material which has not undergone the process of adaptation to *in vitro* culturing. In contrast to monolayer systems, our 3D model exhibited a high degree of morphological similarity to the patient's tumor histology, suggesting that it could more faithfully replicate the clinical scenario than standard culture methods. Moreover, on 2D supports, cancer cells may be overtaken by infiltrating stroma, e.g. cancer-associated fibroblasts and fibroblast-like cells, requiring the use of selective media, specific growth factors or ad hoc culturing techniques. Conversely, our 3D system provided the necessary stimuli to enrich the liposarcoma cell fraction over the stromal counterpart. More importantly, 2D culturing of near-patient material induces an *in vitro* adaptation process that may end up in cancer cells losing their characteristic markers (Fan et al., 2012; Talasila et al., 2013). In our study, 3D-cultured cancer cells preserved both *MDM2* amplification and gene expression associated with liposarcoma pathogenesis and aggressiveness (Benassi et al., 2001; Gogou et al., 2012; Guo et al., 2008; Pazzaglia et al., 2004; Stratford et al., 2011; Somarelli et al., 2016), suggesting that this *in vitro* model could facilitate the identification of disease-specific biomarkers. We then used 3D-enriched cancer cells to carry out drug activity screening. Both treatment schedules exerted a cytotoxic effect on the liposarcoma culture. The patient showed no evidence of disease after undergoing three cycles of treatment with the epirubicin plus ifosfamide combination. However, a longer follow up is needed to see whether a correlation exists between the response of the patient and the *in vitro* data. There is also a possibility that the disease eradication was a result of the surgical treatment. Although there is still no acknowledged indication for the use of trabectedin in this liposarcoma subtype, clinical trials on the drug are currently ongoing. Our work also provides a rationale for further exploring the effects of trabectedin in DDLPS as its anti-tumoral activity was comparable with that of epirubicin plus ifosfamide (Demetri et al., 2009; Petek et al., 2015). While the cytotoxicity was similar, epirubicin plus ifosfamide induced a higher apoptotic cell death compared to trabectedin, which is also known to exert its effect by the arrest of the cell cycle (D'Incalci, 2013).

These translational models could also be used to study drug resistance mechanisms, in particular to understand why the majority of DDLPS show limited responsiveness to chemotherapy. Although surgical resection of DDLPS usually involves the excision of a substantial amount of tissue, the material excised from our patient was scant as it was a recurrent lesion. Inoculation of primary cells into mouse models was therefore not feasible and the experimental approach had to be scaled down to accommodate the limited material available. In such settings, the zebrafish model, requiring only a small number of cells per assay, becomes a valuable resource as it allows for the evaluation of complex cancer-related features such as cell migration, extravasation, invasion and metastasis formation (He et al., 2012; Pickart and Klee, 2014; Stoletov and Klemke, 2008). The model also has at least two other important advantages: the transparent body of the embryos permits an easy follow up of injected cells, and clear results can be obtained after only a few days (Konantz et al., 2012). For these reasons we functionally assayed primary cells in zebrafish, successfully engrafting for the first time liposarcoma cells from a near-patient culture into this *in vivo* model. The cells were retained until the end of experiment at 4 dpi and showed progressive migration from the site of implantation. This finding is in line with our *in vitro* functional characterization of these cells which showed extensive extracellular matrix (ECM) remodeling abilities and MMP upregulation when the cells were seeded on collagen scaffolds. This migratory phenotype has been well documented in zebrafish xenografts of malignant cells and is not recapitulated by healthy or non-transformed, fibrotic-derived tissues (Barriuso et al., 2015). In addition, the previously mentioned studies (Bansal et al., 2014; Barriuso et al., 2015; Bentley et al., 2015; Marques et al., 2009; Orlova et al., 2014; Pruvot et al., 2011; Rampazzo et al., 2013) reported that cells derived from healthy tissues and from non-invasive malignant cells are not retained in the zebrafish and tend to disappear a few days after transplantation. Moreover, the *in vivo* behavior of these cells closely resembled that of the local invasive phenotype in the patient, as reported in the pathologist's referral of the excised tumor.

Although this work is limited by the inclusion of only a single primary culture, we provided proof of concept that the 3D and the zebrafish models could be used in translational research studies for biomolecular analyses, drug screenings and tumor invasiveness assays of patient-derived material.

Optimization and validation of these models through the culturing and xenografting of additional primary specimens is required, especially in order to explore the usefulness of these systems to study different tumor subtypes maintaining their heterogeneity. Finally, the use of the zebrafish may be exploited to compare the *in vivo* behavior of primary cells cultured in 3D or in monolayer systems, and to test the efficacy of drugs on primary cells engrafted *in vivo*.

## MATERIALS AND METHODS

### Collagen scaffold synthesis and characterization

All chemicals were purchased from Sigma-Aldrich (St. Louis, MO, USA). The collagen scaffolds were synthesized as follows (Minardi et al., 2014): an acidic suspension of 1 wt% bovine type I collagen was prepared and precipitated to pH 5.5. The material was crosslinked with 1wt% 1,4-butanediol diglycidyl ether (BDDGE) to stabilize the collagen matrix and control porosity. The final monolithic scaffold was generated through a freeze-drying process. An established freezing and heating ramp yielded scaffolds with optimal levels of pore interconnectivity, orientation and porosity. All scaffolds were sterilized by immersion in 70% ethanol for 1 h, followed by three washes in sterile Dulbecco phosphate buffered saline (DPBS) (Life Technologies, Carlsbad, CA). The scaffolds were imaged by Scanning Electron Microscopy (SEM) as follows. The samples were washed three times with 0.1 M sodium cacodylate buffer pH 7.4, fixed in 2.5% glutaraldehyde in 0.1 M sodium cacodylate buffer pH 7.4 for 2 h at 4°C and washed again in 0.1 M sodium cacodylate buffer pH 7.4. Samples were then dehydrated in a graded series of ethanol for 10 min each, dried in a desiccator overnight and sputter-coated with platinum. Images were acquired with Nova NanoSEM scanning electron microscope (FEI, Hillsboro, OR).

### Patient history

A 53-year-old man with an abdominal liposarcoma previously operated on in another hospital was seen at our institute. A contrast-enhanced chest-abdomen CT scan revealed the presence of three residual tumor nodules of about 12, 15 and 21 mm in diameter in the retroperitoneal pelvic space, with infiltration of muscular fascia and perilesional tissue. The patient refused chemotherapy before surgery and underwent tumor excision with radiofrequency dissector. Histological analysis of the surgical specimen (10.5×7×3 cm), composed of four contiguous nodular lesions of 3.5, 3, 1.2 and 0.5 cm, revealed dedifferentiated grade 3 liposarcoma according to the French Federation of Cancer Centers Sarcoma Group (FNCCL) grading system. Large areas of necrosis and numerous mitoses were present. Cancer cells were positive for *MDM2* amplification and desmin, with focal actin expression, and negative for miogenin. The patient underwent three cycles of adjuvant chemotherapy with epirubicin plus ifosfamide and in May 2016, 9 months after the end of treatment, radiological evaluation showed no evidence of disease.

### Isolation of primary liposarcoma cells

Tumor specimens were obtained from a surgically resected retroperitoneal lesion. The surgical material was analyzed and selected by a pathologist experienced in sarcoma and processed within 3 h of removal. The specimen was washed twice in sterile phosphate buffered saline (PBS) supplemented with 10% penicillin/streptomycin and disaggregated into 1-2 mm^3^ pieces with sterile surgical blades. The fragments obtained were incubated with 2 mg/ml of collagenase type I (Millipore Corporation, Billerica, MA) at 37°C under stirring conditions. The enzymatic digestion was stopped after 2 h by adding DMEM supplemented with 10% fetal bovine serum, 1% glutamine and 10% penicillin/streptomycin. The cell suspension was then filtered with 100 µm sterile mesh filters (CellTrics, Partec, Münster, Germany). Cells were counted and seeded in standard monolayer cultures at a density of 80,000 cells/cm^2^ or in collagen-based scaffolds at a density of 500,000 cells/57 mm^3^. All cells were maintained in complete DMEM medium at 37°C in a 5% CO_2_ atmosphere. All downstream analyses were performed after 3 weeks of culture in either setting.

The study was reviewed and approved by the Local Ethics Committee and performed in accordance with the principles of Good Clinical Practice and the Helsinki declaration. The patient provided written informed consent to take part in the study.

### Immunohistochemical analysis

The 3D constructs were fixed in 10% neutral buffered formalin for 24 h and dehydrated in a graded series of ethanol. Samples were then embedded in paraffin, sliced with a rotating microtome (Leica Biosystems) at a thickness of 5 µM and mounted on Superfrost Plus microslides (Thermo Fisher Scientific, Waltman, MA). Hematoxylin and eosin (H&E) staining was performed to evaluate scaffold architecture and cell morphology and distribution. *MDM2* amplification was detected by FISH (Vysis MDM-2/CEP12 dual color FISH Probe Kit) according to the manufacturer's instructions. For monolayer cultures, 100,000 cells were cytospinned onto glass slides and *MDM2* amplification evaluated by FISH.

### Cell recovery from scaffolds and downstream characterization

Cells were recovered from the 3D constructs by disaggregating the scaffolds into 1-2 mm^3^ pieces with sterile surgical blades followed by enzymatic digestion in 2 mg/ml of collagenase type I (Millipore Corporation) for 1 h at 37°C under stirring conditions. The cell suspension was then filtered with 100 µm sterile mesh filters (CellTrics, Partec).

### Quantitative real-time reverse transcriptional-PCR (qRT-PCR)

For RNA extraction, cell-seeded scaffolds were fragmented into small pieces and 2D culture cells were collected by tripsinization. Total mRNA was isolated using TRIzol Reagent (Invitrogen) following the manufacturer's instructions. Five hundred nanograms of RNA were reverse-transcribed using the iScript cDNA Synthesis Kit (BioRad, Hercules, CA). Real-Time PCR was performed on the 7500 Real-Time PCR System using the TaqMan gene expression assay mix (Applied Biosystems, Foster City, CA). Amplification was performed in a final volume of 20 µl containing 2× Gene expression master Mix, 2 µl of cDNA in a total volume of 20 µl. The stably expressed endogenous β-actin, β-2-microglobulin, HPRT and 18S rRNA were used as reference genes. The following markers were analyzed: ALDH1A1, CDH1, CTNNB1, MMP2, MMP9, SNAI2, GAPDH. The amount of transcripts was normalized to the endogenous reference genes and expressed as *n*-fold mRNA levels relative to a calibrator using a comparative threshold cycle (Ct) value method (ΔΔCt). RNA extracted from the tumor sample was used as calibrator.

### Drug testing

For drug assessment, 3D-recovered cells were seeded in 96-well plates at a density of 80,000 cells/cm^2^. Cells were allowed to recover for 3 days and then treated with the following schedules: epirubicin 2 µg/ml plus ifosfamide 100 µM or trabectedin 17 ng/ml, according to the plasma peak levels of each compound (Highley et al., 2015). Medium was changed after 48 h and replaced with fresh complete DMEM. Survival percentages were assessed after a 24 h washout by MMT assay (Sigma-Aldrich) in accordance with the manufacturer's instructions.

### TUNEL assay

Fragmented DNA generated in response to apoptotic signals was detected by the terminal deoxynucleotidyl transferase (TdT) nick end labeling (TUNEL) assay. After each treatment schedule, cells were washed twice with PBS, fixed by incubation in 1% formaldehyde on ice for 15 min and in 70% ice cold ethanol for 1 h. Cells were then washed twice in PBS, permeabilized in 0.1% Triton-X100 for 5 min and incubated in 50 µl of solution containing TdT and FITC conjugated dUTP deoxynucleotides 1:1 (Roche Diagnostic GmbH, Mannheim, Germany) in a humidified atmosphere for 90 min at 37°C in the dark. Nuclei were counterstained with ProLong Gold antifade reagent with DAPI (Invitrogen, Life Technologies) and the samples were analyzed by inverted fluorescence microscopy.

### *In vivo* experiments with the zebrafish model

#### Zebrafish maintenance and cancer cell engraftment

Zebrafish and embryos were raised, staged and maintained according to standard procedures in compliance with local animal welfare regulations and the EU Animal Protection Directive 2010/63/EU. The transgenic line Tg(Kdrl:mCherry) was used for this study and was kindly provided by Prof. Schulte-Merker, Institute for Cardiovascular Organogenesis and Regeneration, Faculty of Medicine, Westfälische Wilhelms-Universität Münster (WWU). 0.2 mM of *N*-phenylthiourea (PTU; Sigma) was used to prevent pigment formation from 1 day post-fertilization (dpf) onward.

#### Embryo preparation and tumor cell implantation

After 3 weeks of monolayer culture, liposarcoma cells were labeled with CellTrace™ CFSE Cell Proliferation Kit (Life Technologies) according to the manufacturer's instructions, with a final dye concentration of 5 µM. The labeled cell suspension was further re-suspended in 3 mg/ml of growth factor-reduced Matrigel™ (BD Biosciences, Franklin Lakes, NJ, USA) diluted in ice cold sterile DPBS (Gibco, Life Technologies) and loaded into borosilicate glass capillary needles (1 mm outer diameter×0.78 mm inner diameter; Harvard Apparatus) within 3 h of cell harvest, to a final concentration of 2.5×10^5^ cells/µl. Two-day-old (2 dpf) zebrafish embryos were anesthetized with 0.003% tricaine (Sigma) and positioned on a 10 cm Petri dish coated with 1% agarose dissolved in eggwater (demineralized water containing 60 μg/ml of seasalt). 50-400 manually counted cells were injected in the embryonic heart cavity using a Pneumatic Pico pump and a micromanipulator (WPI), and 100 embryos were implanted with cancer cells. After implantation with cancer cells, the zebrafish embryos (including non-implanted controls) were maintained at 34°C as a compromise between the optimal temperature requirements for fish and mammalian cells (Haldi et al., 2006). Fluorescent image acquisition was performed using a Leica MZ16FA stereo microscope (Leica Microsystems GmbH, Wetzlar, Germany). Up to 400 implantations were manually achieved per hour, with survival rates of *>*80% up to the fourth day post-implantation (dpi). Images were further analysed with the software ImageJ (Rasband, W.S., ImageJ, U. S. National Institutes of Health, Bethesda, Maryland, USA, http://imagej.nih.gov/ij/, 1997-2016).

### Statistical analysis

At least three independent biological replicates were performed for each experiment. Data are presented as mean±standard deviation (s.d.), or mean±standard error (s.e.), as stated, with *n* indicating the number of replicates. For *in vitro* and *in vivo* data, differences between groups were assessed by a two-tailed Student's *t*-test and accepted as significant at *P*<0.05.
