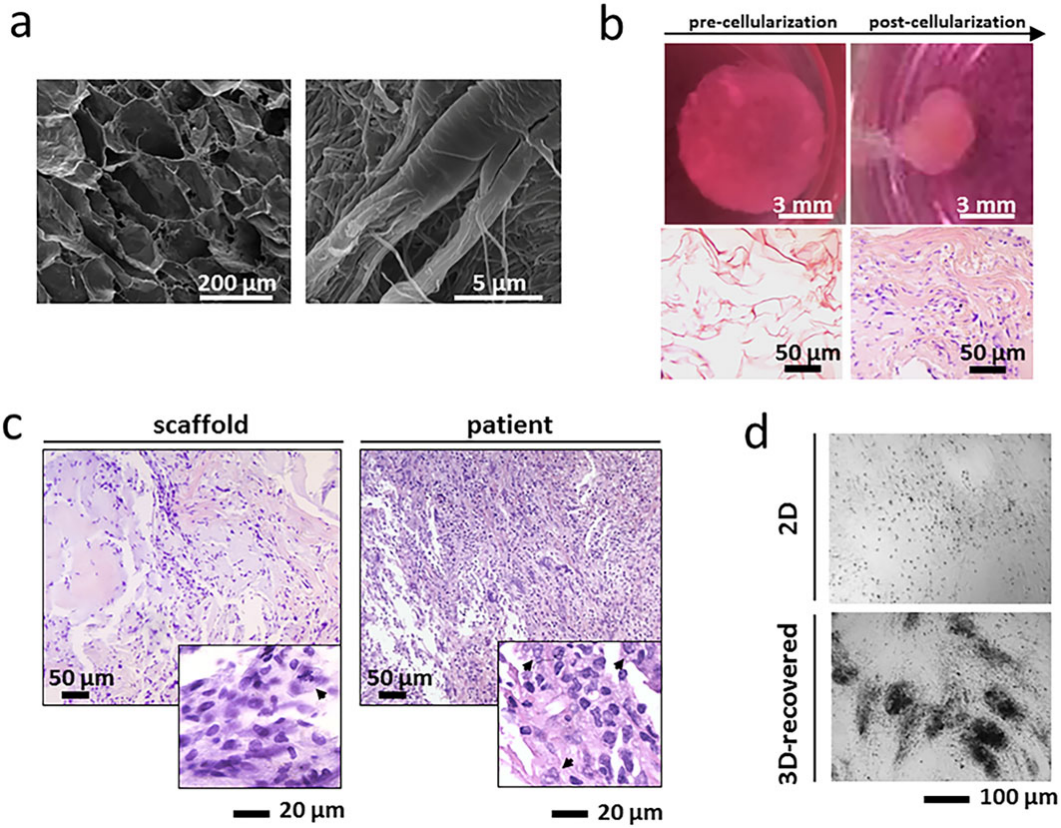# CORRECTION: Innovative approaches to establish and characterize primary cultures: an *ex vivo* 3D system and the zebrafish model

**DOI:** 10.1242/bio.023911

**Published:** 2017-02-08

**Authors:** Chiara Liverani, Federico La Manna, Arwin Groenewoud, Laura Mercatali, Gabri Van Der Pluijm, Federica Pieri, Davide Cavaliere, Alessandro De Vita, Chiara Spadazzi, Giacomo Miserocchi, Alberto Bongiovanni, Federica Recine, Nada Riva, Dino Amadori, Ennio Tasciotti, Ewa Snaar-Jagalska, Toni Ibrahim

The previously posted version of Biology Open article doi:10.1242/bio.022483, published ahead of print on 28 November 2016, has been corrected to credit Dr Silvia Minardi (Department of Regenerative Medicine, Houston Methodist Research Institute, Houston, TX, USA) with regards the collagen scaffolds used in the work.

The following changes have been incorporated in the final issue version of the article:

 1. In agreement with Dr Minardi, the right-hand panel of Fig. 1A has been replaced. The corrected figure is below. There are no changes to the figure legend, which is accurate.

 2. The sentence “SEM evaluation revealed the formation of 14.682-μm-thick collagen boundaries and confirmed that the typical D-bands of type I collagen were preserved” related to the previous version of Fig. 1A, and so has been deleted from the section Results – Establishment of the *ex vivo* 3D tumor model.

 3. The reference Minardi et al., 2014 has been added to the sentence “The collagen scaffolds were synthesized as follows (Minardi et al., 2014): an acidic suspension of 1 wt% bovine type I collagen was prepared and precipitated to pH 5.5.” in the section Materials and Methods – Collagen scaffold synthesis and characterization.

 4. The Acknowledgements section now reads as follows: “The authors thank Dr Silvia Minardi for fabricating the collagen scaffolds utilized in this manuscript, and Cristiano Verna for editorial assistance.”

 5. The following reference has been added:

**Minardi, S., Sandri, M., Martinez, J. O., Yazdi, I. K., Liu, X., Ferrari, M., Weiner, B. K., Tampieri, A., Tasciotti, E.** (2014). Multiscale patterning of a biomimetic scaffold integrated with composite microspheres. *Small* **10**, 3943-3953.

These changes do not affect the conclusions of the paper.

The authors apologise to the readers for any confusion that these changes might have caused.

**Figure:**